# Functional and Probiotic Attributes of an Indigenous Isolate of *Lactobacillus plantarum*

**DOI:** 10.1371/journal.pone.0008099

**Published:** 2009-12-01

**Authors:** Jai K. Kaushik, Ashutosh Kumar, Raj K. Duary, Ashok K. Mohanty, Sunita Grover, Virender K. Batish

**Affiliations:** 1 Animal Biotechnology Centre, National Dairy Research Institute, Karnal, India; 2 Molecular Biology Unit, Dairy Microbiology Division, National Dairy Research Institute, Karnal, India; Universita di Sassari, Italy

## Abstract

**Background:**

Probiotic microorganisms favorably alter the intestinal microflora balance, promote intestinal integrity and mobility, inhibit the growth of harmful bacteria and increase resistance to infection. Probiotics are increasingly used in nutraceuticals, functional foods or in microbial interference treatment. However, the effectiveness of probiotic organism is considered to be population-specific due to variation in gut microflora, food habits and specific host-microbial interactions. Most of the probiotic strains available in the market are of western or European origin, and a strong need for exploring new indigenous probiotic organisms is felt.

**Methods and Findings:**

An indigenous isolate Lp9 identified as *Lactobacillus plantarum* by molecular-typing methods was studied extensively for its functional and probiotic attributes, viz., acid and bile salt tolerance, cell surface hydrophobicity, autoaggregation and Caco-2 cell-binding as well as antibacterial and antioxidative activities. Lp9 isolate could survive 2 h incubation at pH 1.5–2.0 and toxicity of 1.5–2.0% oxgall bile. Lp9 could deconjugate major bile salts like glycocholate and deoxytaurocholate, indicating its potential to cause hypocholesterolemia. The isolate exhibited cell-surface hydrophobicity of ∼37% and autoaggregation of ∼31%. Presence of putative probiotic marker genes like mucus-binding protein (*mub*), fibronectin-binding protein (*fbp*) and bile salt hydrolase (*bsh*) were confirmed by PCR. Presence of these genes suggested the possibility of specific interaction and colonization potential of Lp9 isolate in the gut, which was also suggested by a good adhesion ratio of 7.4±1.3% with Caco-2 cell line. The isolate demonstrated higher free radical scavenging activity than standard probiotics *L. johnsonii* LA1 and *L. acidophilus* LA7. Lp9 also exhibited antibacterial activity against *E. coli*, *L. monocytogenes, S. typhi*, *S. aureus* and *B. cereus*.

**Conclusion:**

The indigenous *Lactobacillus plantarum* Lp9 exhibited high resistance against low pH and bile and possessed antibacterial, antioxidative and cholesterol lowering properties with a potential for exploitation in the development of indigenous functional food or nutraceuticals.

## Introduction

Human digestive tract houses numerous bacterial species of diverse types. Among them, lactobacilli and bifidobacteria, which can ferment a variety of nutrients primarily into lactic acid or other by-products, constitute a major functionally related group of enteric organisms. Whole genome sequencing has revealed their complex nutritional requirements due to lifestyle adaptation [1]–[4]. A number of lactic acid bacterial (LAB) species have evolved symbiotically with animal species they harbor. The gut microbiota includes a very important group of friendly bacteria of which lactobacilli and bifidobacteria are the two key members which have been implicated in a number of health promoting functions that affect general health and well-being of the host. These microorganisms are called probiotics, which means “for life” [5]. World Health Organization (WHO) has defined probiotics as live microorganisms which when administered in adequate amounts confer a health benefit on the host [6]. There are numerous probiotic genera and species including lactobacilli and bifidobacteria. These organisms favorably alter the intestinal microflora balance, promote intestinal integrity and mobility, inhibit the growth of harmful bacteria and increase resistance to infection [7] and should possess the properties like survival in the gastrointestinal (GI) tract, persistence in the host, and proven safety for consumer [8], [9]. The survivability and colonization in the digestive tract are considered critical to ensure optimal functionality and expression of health promoting physiological functions by probiotics. To survive in the gut, the organisms must be tolerant to low pH and bile toxicity prevalent in the upper digestive tract. For colonization, they should exhibit good surface hydrophobicity and aggregation properties [10], [11]. Functionally, they may neutralize the effect of pathogens by inhibiting the toxin action or their production [12], express bacteriocins and inhibit the binding of pathogens with mucosal surface [13]–[17]. They may also show antioxidative and immunomodulatory activities [18], [19].

*In vitro* and animal model studies have indicated that probiotic organisms can enhance both specific as well as non-specific immune response by activating macrophages, altering cytokine expression, increasing NK cell activity, and increasing the immunoglobulin level [18]–[22]. These organisms also colonize the urogenital tract of females, preventing yeast infection and growth of pathogenic bacteria [14], [23], [24]. Some probiotic bacteria produce bacteriocins, which act as natural antibiotics to kill undesirable microorganisms. Topical and oral use of *L. acidophilus* can prevent yeast infection caused by Candida overgrowth. The traveler's diarrhea caused by pathogenic bacteria can also be reduced by the preventive use of probiotics [25]. More recently it has been reported that probiotics can play a very important role in the prevention of respiratory tract infections [26].

Lactic acid bacteria play a very important role as starters in the production of fermented health foods since they are food-grade organisms and are generally regarded as safe (GRAS) [20]. Selected strains of *Lactobacillus* spp. and *Bifidobacterium* spp. are increasingly being used as viable probiotics into various food products [27].

After screening a large number of lactobacilli based on their bile-tolerance and adherence-potential using Caco-2 cell line model, a promising isolate was selected in this study. The isolate Lp9 was identified as *Lactobacillus plantarum* by genus and species-specific PCR as well as 16S rRNA sequencing. The selected culture was subjected to characterization for functional and probiotic attributes. *L. plantarum* is a versatile organism with broad-lifestyle and commonly encountered in dairy, meat, and plants as well as in GI tract of humans and animals. *L. plantarum* has a long history of safe use for preparation of fermented foods and it is also an important member of the GI tract microflora. A number of ethnic as well as commercial probiotic preparations are available in the market based on *L. plantarum* [9]. The survival and potential to deliver health benefits differ markedly among various strains of *L. plantarum*. Genomic studies indicated the presence and absence of different DNA regions, including those encoding production of bacteriocin, exopolysaccharides and genes involved in sugar metabolism, in various strains of *L. plantarum* [28]. These genetic variations could affect the adaptability and survivability of the organism under a range of environmental niches, and thus also impacting their potential to serve as a probiotic organism.

To determine the suitability of the Lp9 isolate for exploitation as a probiotic, particularly for application in Indian population, we studied the probiotic properties like pH and bile salt tolerance and bile salt hydrolase (Bsh) activity, cell surface hydrophobicity and autoaggregation, antibacterial and antioxidative activities as per FAO/WHO guidelines [29]. The isolate showed promising results in comparison to probiotic species like *L. johnsonii* LA1 and *L. acidophilus* LA7. The genes encoding putative cell surface binding factors like mucus-binding protein (Mub) and fibronectin-binding protein (Fbp) involved in the cellular interaction, and hence causing a prolonged retention of microorganisms in the host's digestive system and also helping the probiotic organism to competitively inhibit the pathogens, were also detected by PCR assays. In this paper, we report the identification of an indigenous isolate of *L. plantarum* and its various probiotic and functional properties.

## Results

### Screening and Identification of Indigenous Lactobacilli Isolates

A total of 100 typical lactobacilli isolates recovered from 35 samples (comprising 25 human fecal and 5 each of human milk and raw buffalo milk collected from different sources) on BCP-MRS plates were initially selected for screening the potential probiotic lactobacilli isolates. The typical colonies of these lactobacilli isolates were randomly selected from BCP-MRS plates based on their morphological characteristics on microscopic examination after Gram's staining and catalase negative reaction. 16S rRNA based genus–PCR carried out by employing the primer pair LbLMA1/R-161 (Table 1) resulted in the amplification of the desired amplicon of 250 bp specific for the genus *Lactobacillus* for only 33 of the isolates. Out of these, 24 were from human fecal samples, eight from raw buffalo milk and one from human milk. Three standard probiotic cultures namely *L. johnsonii* LA1, *L. acidophilus* LA7 and *L. plantarum* CSCC5276 (also designated as NCDO 82 or VTT E-71034) [30] were used as positive controls (data not shown) in the PCR assays.

**Table 1:** List of primer pairs used in PCR amplification.

Primer pair (Forward/Reverse)	Primer sequence (5′→3′)	Target gene	Amplicon length (bp)	Anneal. Temp. (°C)	Ref.
LbLMA-1/R-161	ctcaaaactaaacaaagtttc cttgtacacaccgcccgttca	16S rRNA	250	55	[85]
Lpla3/Lpla2	attcatagtctagttggaggt cctgaactgagagaatttga	16S rRNA specific-*L. plantarum*	248	60	[88]
LaBSHF/R	ttcatcgtttgcagttgctc gagctgtagcgtcatgtgga	Bile salt hydrolase	196	56	This study
LjBSHAF/R	atagtcgcgggttagggact catctgttccctttggctgt	Bile salt hydrolase	171	56	This study
LjBSHBF/R	tccttggggtgtaggaactg cctttgatcatggcaacaga	Bile salt hydrolase	168	56	This study
LgBSHF/R	tccatcccttttgcttgttc gttccaggcgaacctgataa	Bile salt hydrolase	220	56	This study
LpBSHF/R	atcaccgctacattggttgg agtccgcccattcctctact	Bile salt hydrolase	231	56	This study
LpBSHF1/R1	atgtgtactgccataacttat ttagttaactgcatagtattg	Bile salt hydrolase	975	56	This study
LpMubD1F/R	atgcgtatcggacggctgatac aacagcctcaaaacgaccagtc	MUB-domain of Mub protein	425, 1050	55	This study
LpMubNF/R	tacattcaagatgcagcgggcaa ccaccctgatcagttaacgtgcc	N-terminal of Mub protein	1640	55	This study
LpFBPF/R	gtcctttgatggtttatttaccc agaagtatgcggcgagattcgc	Fibronectin binding protein	1500	50	This study

These isolates were again screened for the presence of bile salt hydrolase (*bsh*) gene, since Bsh enzyme is considered as an important probiotic marker that helps organisms resist toxic bile salt environment in the GI tract. The *bsh* genes of acidophilus group and *L. plantarum* were targeted to design primers since they are known to represent major groups among the lactobacilli residing in the gut. Two sets of *bsh* primers targeted against *L. johnsonii* (LjBSHAF/R and LjBSHBF/R) and one pair against each of *L. acidophilus* (LaBSHF/R) and *L. gasseri* (LgBSHF/R) did not result in any amplification of the expected size of the PCR product. However, at least 10 of the isolates showed the amplification of an expected PCR product of size 231 bp when assayed using *L. plantarum* specific primers (LpBSHF/R).

**Figure 1:**
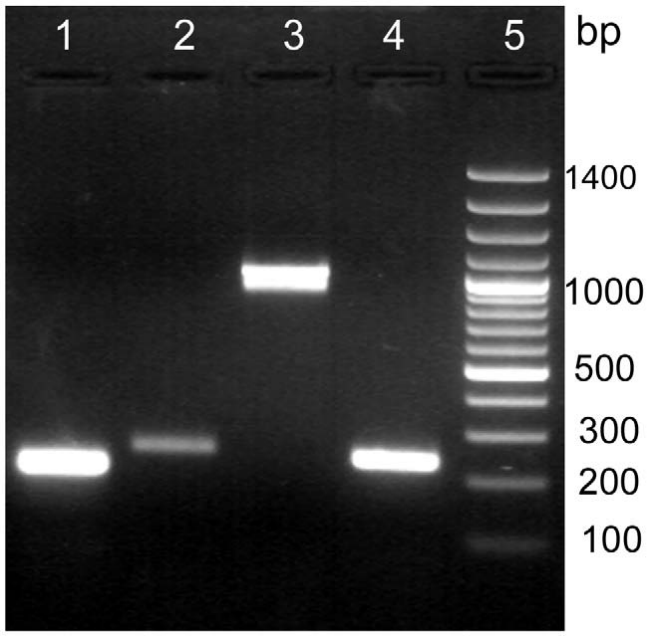
Identification of Lp9 isolate by molecular-typing methods. Molecular typing of Lp9 isolate by using LbLMA1/R161genus-specific (lane 1) and species-specific primers (lane 2) and *L. plantarum* specific primers for amplification of full length 975 bp *bsh* gene (lane 3) and partial 231 bp *bsh* gene (lane 4) by PCR. Lane 5–100 bp DNA ladder.

These isolates were again subjected to 16S rRNA based species-specific PCR assays by employing *L. plantarum* specific primers Lpla3/Lpla2 (Table 1). The amplification of a product of 248 bp in case of all the 10 isolates reconfirmed them to be *L. plantarum.* Figure 1 shows the fragments amplified from genomic DNA of Lp9 by using genus-specific, species-specific and *bsh* gene specific primers in the PCR assays.

The adherence of the putative probiotic organism in the GI tract is most crucial for an extended residence time in the host. Therefore, all the 10 isolates were subjected to *in vitro* adherence test by using human carcinoma cell line Caco-2, which has been widely used as a model system to study the interaction of bacteria with human enterocytes [31]–[33]. On qualitative evaluation, the isolate Lp9 was observed to be most strongly adhering with Caco-2 cells. The adhesion ratio of Lp9 isolate with Caco-2 cell culture was estimated to be 7.4±1.3%, which was comparable with the reported adhesion values of 6.7±1.4% and 8.5±2.5% of *L. plantarum* ATCC 8014 [31] and *L. plantarum* 122E [33] with Caco-2 cells, respectively. These results showed that the adhesion potential of Lp9 with Caco-2 cell culture was comparable or significantly greater than several other dairy cultures and a number of probiotic strains with reported health effects [31]–[33]. Some well adopted commercial strains of lactobacilli used as dairy cultures or probiotic adjuncts showed adhesion as high as 12.6–14.4% on the one hand and as low as 2.6–4.8% on the other hand [31]. Although Lp9 originated from buffalo milk, yet its good binding with the human cell line suggested that the isolate could also adhere and persist in the human gut. These *in vitro* studies suggested the suitability of Lp9 for detailed characterization of its various functional and probiotic properties as per FAO/WHO guidelines [29].

Finally, sequencing and analysis of 1314 bp long 16S rRNA gene of Lp9 isolate was carried out. All the top hits in the BLAST search using 16S rRNA sequence as a query revealed highest sequence identity with the *L. plantarum* species with an E-value of 0.0 and sequence coverage of 99%. The 16S rRNA sequence has been deposited in the Genbank database (Accession no. GQ337858). *L. plantarum* Lp9 culture has been deposited in the National Collection of Dairy Cultures (NCDC) at National Dairy Research Institute, India (Accession no. NCDC 344).

### Probiotic Attributes of Lp9 Isolate of *L. plantarum*

The indigenous isolate Lp9 after its confirmation as *L. plantarum* by genus and species-specific PCR assays as well as by 16S rRNA sequencing was subjected to a battery of tests as per FAO/WHO guidelines [29] discussed in the following sections.

#### Acid tolerance

The Lp9 isolate was subjected to low pH prevalent in the stomach for 2 h at 37°C. The initial log (cfu/ml) of 8.9 decreased to 7.3 and 8.4 at pH 1.5 and 2.0, respectively. The marginal decrease in the log (cfu/ml) value at low pH indicated the good tolerance of Lp9 against acidic conditions prevalent in the stomach.

#### Bile salt tolerance and Bsh activity

The initial log (cfu/ml) of 8.5 of Lp9 isolate got decreased by 1-log cycle after 2 h incubation at 37°C in MRS broth containing 1.5–2.0% bile, which was about 5–6 times more concentrated than bile present in human intestine. These results indicated the survival potential of Lp9 in the presence of toxic bile salts.

Some strains of *L. casei* have been observed to grow in the presence of 0.5% bile without bile salt hydrolyzing ability [34], suggesting that bile tolerance may not necessarily be an outcome of Bsh production. Therefore, to observe whether Lp9 produced a functional Bsh enzyme for the deconjugation of bile salts, the Lp9 isolate was streaked on the MRS agar supplemented with 0.3–0.5% concentration of bile salts like sodium deoxycholate (DCA), sodium taurodeoxycholate (TDCA) and sodium glycocholate (GCA). Growth of Lp9 and a zone of salt precipitation around the colonies at both the concentrations could be observed on plates supplemented with these bile salts. Lp9 could grow in the presence of GCA and TDCA at concentration as high as 0.5%, which suggested that Lp9 produced Bsh activity specific to GCA and TDCA hydrolysis. On the other hand, the precipitation zone around spotted culture on DCA plate could be due to precipitation of DCA as a result of lowering of pH by Lp9. DCA is a deconjugated bile salt and hence there cannot be any deconjugation reaction. These results indicated that Lp9 can survive not only the toxicity of these salts but also can carry out Bsh mediated deconjugation of GCA and TDCA. The PCR amplification using species-specific primers (LpBSHF1/R1) designed against full length *bsh* gene resulted in a product of 975 bp (Figure 1), which was consistent with the size of *bsh* gene in *L. plantarum*. These results confirmed the observed bile salt deconjugation activity of Lp9 isolate.

#### Cell surface hydrophobicity

The percent cell surface hydrophobicity of Lp9 as well as control strains like *L. johnsonii* LA1 and *L. acidophilus* LA7 determined by using two hydrocarbons namely n-hexadecane and xylene are listed in Table 2. The hydrophobic values obtained for Lp9 in the presence of n-hexadecane or xylene were almost similar (37.1–37.7%) within the experimental errors, while for *L. johnsonii* LA1 and *L. acidophilus* LA7, the observed values were ∼47% and ∼57–58%, respectively, under identical conditions. *L. plantarum* from goat showed surface hydrophobicity of 47–69% depending upon the solvent used [35]. Cell surface hydrophobicity of some strains of *L. johnsonii* and *L. acidophilus* has been reported as high as 23–88% and 74–95%, respectively [36]. However, some strains of lactobacilli including those from *L. acidophilus* group, which also includes *L. johnsonii*, showed surface hydrophobicity as low as 2–5% [36], [37]. The large differences in the cell surface hydrophobicity could be due to variation in the level of expression of cell surface proteins among strains of a species as well as due to environmental conditions which could affect the expression of surface proteins [9], [38], [39].

**Table 2:** Cell surface hydrophobicity of Lp9 and other probiotic cultures.

	Hydrophobicity (%)		
Culture	n-hexadecane	Xylene	Aggregation (%)
Lp9	37.7±1.3^a^	37.1±3.9	31.0±1.0
*L. johnsonii* LA1	46.8±1.5	45.8±0.3	40.4±0.4
*L. acidophilus* LA7	56.7±0.5	58.2±0.5	46.5±2.0

a All the values are shown as mean±sd (standard deviation) of 3–5 replicate experiments.

#### Cellular autoaggregation

To evaluate the cell aggregation potential of Lp9, the cellular autoaggregation was measured as described in the method section [10], [38]. Table 2 lists the percent cell autoaggregation of Lp9, *L. acidophilus* LA7 and *L. johnsonii* LA1 cultures carried out under identical conditions. *L. acidophilus* LA7 showed highest cell autoaggregation (46.5%) followed by *L. johnsonii* LA1 (40.4%) and Lp9 (31%); a similar trend was also obtained for the cell surface hydrophobicity of these cultures. These results indicated the capability of Lp9 to self-aggregate, although to a lesser extent than other two standard probiotic cultures.

#### Antibacterial activity

Antibacterial activity of Lp9 was studied against common pathogens like *Escherichia coli*, *Staphylococcus aureus*, *Listeria monocytogenes, Salmonella typhi* and *Bacillus cereus*. Table 3 shows the antibacterial activity of Lp9 as a zone of inhibition against these pathogens. The extracellular overnight spent supernatant of Lp9 exhibited varying zones of inhibition from 14 mm to 29 mm depending upon the tested pathogen. *B. cereus* was most sensitive with 29 mm diameter of zone of inhibition while *S. aureus* and *E. coli* showed least sensitivity with a zone of 14–16 mm. On the other hand, *L. monocytogenes* and *S. typhi* showed inhibition zones of 20 mm. The standard probiotic culture *L. plantarum* (CSCC5276) also showed similar trend of inhibition of various pathogens. The neutralized extracellular spent supernatant resulted in a complete loss of antibacterial activity against all the tested strains of pathogens. These results suggested that the observed antibacterial activity against the tested pathogens could be due to acidic condition produced by Lp9.

**Table 3:** Antibacterial activity of Lp9 isolate against various pathogens.

	Diameter of zone of inhibitiona,b (mm) by	
Indicator pathogenic cultures	*L. plantarum* (Lp9)	*L. plantarum* (CSCC5276)
*B. cereus*	29	30
*E. coli*	16	20
*Listeria monocytogenes*	20	22
*Salmonella typhi*	20	20
*Staphylococcus aureus*	14	15

a Diameter of the well −7 mm.

b The values shown are mean of three replicates.

#### Antioxidative activity

**Figure 2:**
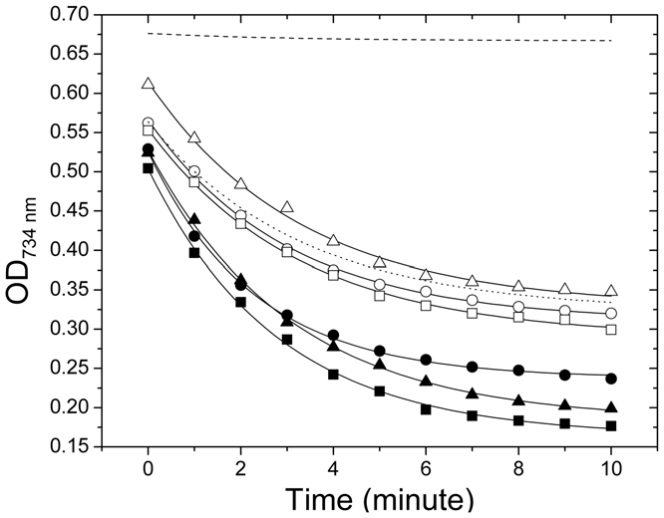
Antioxidative activities of Lp9 and other probiotics. The free radical scavenging activity measured by ABTS method in the extracellular (open) and intracellular (solid) extracts of the Lp9 isolate (circle), *L. johnsonii* LA1 (triangle) and *L. acidophilus* LA7 (square). Phosphate buffer saline (dash line) and MRS broth (dotted line) were used as controls for intracellular and extracellular extracts, respectively. The solid continuous curves are the non-linear least-square fit of first order kinetic model given by equation 4 to the experimental data.

Figure 2 shows the rate of scavenging of 2,2′-Azinobis(3-ethylene benzothiazoline) 6-sulphonic acid free radical cations (ABTS^•+^) by Lp9 and two other standard probiotic cultures, *L. johnsonii* LA1 and *L. acidophilus* LA7. All the three cultures showed single phase exponential following apparently first-order kinetics (Figure 2). The first-order kinetic model has been successfully employed for evaluating the rate constants for scavenging of ABTS^•+^ by various plant based antioxidative agents [40]. Table 4 shows the reaction halftime (T_1/2 = _0.69/*k_i_*) for a first-order reaction kinetics, where *k_i_* represent the corresponding rate constant. In this case, the reaction halftime (T_1/2_) was defined as the time at which the concentration of free radicals (ABTS^•+^) decreased to half its initial value. A smaller value of T_1/2_ means that the free radical species were removed at faster rate due to higher antioxidative activity of the culture. A smaller T_1/2_ (95.2±2.8 sec) of the spent supernatant of Lp9 as compared to those of *L. acidophilus* LA7 (T_1/2_ = 115.9±4.1 sec) and *L. johnsonii* LA1 (T_1/2_ = 127.7±3.5 sec) suggested that Lp9 produced significantly higher antioxidative activity as compared to other cultures. All these values of T_1/2_ also included contribution from MRS broth which also showed strong antioxidative activity (T_1/2_ = 136.6±8.3 sec). Therefore, the contribution of various competing components was calculated by assuming the scavenging of ABTS^•+^ by antioxidative species produced by the culture and the MRS medium in a parallel first-order reaction. In this scheme rate constants follow the relationship given in equation 1 [41].
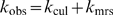
(1)where *k*_obs_ is the experimentally observed apparent rate constant of spent supernatant, which also included the contribution from MRS broth (*k*_mrs_) and the culture (*k*_cul_). By substituting the experimentally observed values of *k*_obs_ and *k*_mrs_ from Table 4 in equation 1, the corresponding value of *k*_cul_ was calculated to be 2.2×10^−3^ sec, 9.1×10^−4^ sec and 3.2×10^−4^ sec for Lp9, *L. acidophilus* LA7 and *L. johnsonii* LA1, respectively; while the corresponding reaction halftimes (T_1/2 = _0.69/*k*_cul_) were estimated to be ∼314 sec, ∼760 sec and ∼2156 sec. These results showed that Lp9 produced much higher concentration of antioxidative species in the extracellular milieu as compared to *L. johnsonii* LA1 and *L. acidophilus* LA7 standard cultures.

**Table 4:** The kinetic parameters for antioxidative potential of Lp9 isolate of *L. plantarum* and other probiotic *Lactobacillus* species.

	Reaction half-time, T_1/2_ (sec)		Rate constant (*k*_obs_) (sec^−1^)	
Culture	Intracellular	Extracellular^a^	Intracellular	Extracellular^a^
Lp9	125.0±4.8^b^	95.2±2.8	5.52×10^−3^	7.25×10^−3^
*L. johnsonii* LA1	135.9±8.3	127.7±3.5	5.08×10^−3^	5.37×10^−3^
*L. acidophilus* LA7	135.9±4.8	115.9±4.1	5.08×10^−3^	5.95×10^−3^
MRS broth	–	136.6±8.3	–	5.05×10^−3^

a The values given for extracellular milieu also include contribution from MRS broth also. For MRS as a blank, *k*_obs =_ *k*_mrs_, while contribution of cultures as *k*_cul_ or reaction halftime (T_1/2_ = 0.69/*k*_cul_) has been deascribed in the results section.

b All the values are shown as mean±sd of three replicate experiments.

Table 4 also showed antioxidative activity of the intracellular milieu. The phosphate buffer saline (PBS) used for cell washing and resuspension of the pellet showed a negligible change in the absorbance as a function of time (Figure 2), indicating that PBS buffer lacked antioxidative activity and the observed decay curves were essentially because of antioxidative property of the cell extract. The intracellular antioxidative activity of Lp9 (T_1/2_ = 125.6±4.8 sec) was observed to be comparable to that of *L. johnsonii* LA1 (T_1/2_ = 135.9±8.3 sec) and *L. acidophilus* LA7 (T_1/2_ = 135.9±4.8 sec). The antioxidative activity in the cell lysate was almost 2.5 fold greater than in extracellular extract of Lp9 (T_1/2_ = 314 sec).

### Mub and Fbp Surface Protein Genes in Lp9 Genome

**Figure 3:**
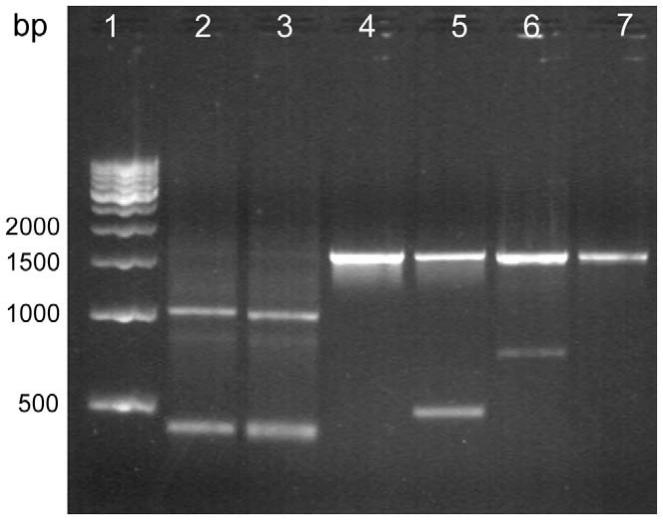
Amplification of *mub* and *fbp* genes by PCR. Genomic DNA of respective strain was used as template DNA and primer sets used in the PCR assay are described in Table 1. Lane 1–500 bp ladder, lane 2 – *MUB* domain of Lp9 (425 bp and 1050 bp), lane 3 – *MUB* domain of *L. plantarum* CSCC5276, lane 4 – N-terminal domain of *mub* in Lp9 (1.6 kbp), lane 5 – N-terminal domain of *mub* in *L. plantarum* CSCC5276, lane 6 – *fbp* in Lp9 (1.5 kbp), lane 7 – *fbp* in *L. plantarum* CSCC5276.

Figure 3 shows agarose gel electrophoresis of the PCR products of *mub* and *fbp* genes amplified by using gene-specific primers. Primer pair LpMubD1F/R, which was designed for repeats of MUB-domains in the *mub* gene, resulted in the amplification of two fragments of sizes 425 bp and ∼1050 bp. The repetitive MUB-domains span approximately 612–615 bp and are located contiguously between bases 2600–6200 of the *mub* ORF of 6660 bp. The repetitive arrangement and high homology among the first two domains might cause amplification of two products of sizes 425 bp and ∼1050 bp because the reverse primer can anneal at the 3′ ends of the first MUB-repeat as well as the second repeat. The primer pair LpMubNF/R targeted against a 1.64 kbp fragment (927–2567 bp), which also comprised a region encoding a 70 amino acid residue MUB-associated domain called Mubad [42] in the N-terminal region of the Mub protein, resulted in the amplification of a unique 1.64 kbp fragment (Figure 3).

Similarly, the presence of another putative cell adhesion protein, the fibronectin-binding protein, was also confirmed by PCR amplification of a ∼1.5 kbp long fragment corresponding to the N-terminal domain by using *fbp* gene-specific primers designed for *L. plantarum*. The standard probiotic culture *L. plantarum* CSCC5276 was used as a positive control. These results confirmed the authenticity of Lp9 identification and its potential for producing surface adhesion proteins required for the extended persistence in the GI tract.

## Discussion

### Survival Potential of Lp9 Isolate in the Gastrointestinal Tract

The Lp9 isolate was observed to survive low pH prevalent in the adult human stomach. The isolate could survive a low pH with only marginal decrease of 0.5-log cycle value at pH 2.0. The human stomach pH is maintained at a value above 2.0. Lankaputhra and Shah [43] also observed strain dependent acid-tolerance in lactobacilli and bifidobacteria at pHs of 1.5–3.0. It has been reported that a minimum daily dose of 10^9^ cfu/day of the strain *L. johnsonii* LA1 is required to modulate non-specific immune response, however the same effect was not observed at a lower cfu/day of 10^6^ [44]. Health effect of probiotic organisms is generally strain dependent and is achieved when the organism is used at 10^8^–10^11^ cfu/day [45]. Our results suggested that Lp9 can tolerate low pH (pH 2.0) without any significant loss in cell count during passage through the stomach.

Lp9 could survive 5–6 fold higher concentration (1.5–2.0% oxgall bile) than the usual bile salt concentration present in human stomach (0.3%). Lp9 could also grow on agar plates containing major constituents of bile salts like DCA, GCA and TDCA at 0.3–0.5% concentration. DCA is deconjugated while TDCA and GCA are conjugated bile acids. Deconjugated bile salts have been reported to be more toxic than conjugated salts [46]–[48]. Lp9 could survive in the presence of deconjugated bile acid DCA and conjugated bile salts like GCA and TDCA. It has been reported that TDCA is more toxic than other bile salts, but it constitutes only minor fraction in the bile. Lp9 could survive low pH as well as the toxicity of bile salts, indicating that the isolate possesses the potential to survive under harsh conditions present in the GI tract.

### GIT-Colonization Potential of Lp9 Isolate

The bacterial adhesion with the gut cells is considered to be an important requirement for a probiotic culture to deliver health effects over an extended period. The surface hydrophobicity of ∼37–38% suggested the adhesiveness of Lp9 isolate. The potential of microorganisms to adhere and colonize in the gut is measured by their cell surface hydrophobicity [49], [50] and aggregation properties, respectively. The cell surface hydrophobicity has been reported from 2% to 95% for different probiotic bacteria [36], [37]. Extensive studies on various strains of *L. johnsonii*, *L. crispatus* and *L. helveticus* showed an enormous variation in the surface hydrophobic properties even among strains of a given species [51]. *L. johnsonii* strain showing hydrophobicity as low as 2% was able to adhere very well with the mucus producing HT29 MTX cells [36]. These observations suggested that hydrophobicity may not be the only criteria but an important component in a complex interplay between specific and non-specific factors which enable a microorganism to bind and persist in the host gut for an extended period. Nevertheless, a similar trend obtained for hydrophobicity and cellular auto-aggregation properties of Lp9 and standard lactobacilli in this study suggests that hydrophobicity may be playing a role in the cellular interaction.

PCR assay revealed the presence of putative fibronectin-binding protein and mucus-binding protein genes in the genome of Lp9. Fbp is an adherence protein [52] and its expression has been shown to modulate microbial adhesion [53]. The genome of *L. plantarum* WCFS1 contains four putative mucus-binding proteins, ORF lp_1643 (∼6660 bp) being the longest with six repeats of the MUB-domain. The mucin-binding Mub proteins may help in the adherence and the persistence of probiotics in the GI tract [42], [52]. Selective amplification of *mub* gene regions corresponding to N-terminal and C-terminal suggests that Lp9 might possess a functional Mub protein, which can provide a specific-binding with host enterocytes. The combination of non-specific interaction mediated through good cell surface hydrophobicity and specific interaction provided by cell surface binding proteins can ensure a good adhesion of Lp9 with the host, which was also suggested by a good binding of Lp9 cells with human enterocytes Caco-2 cell-line.

After adherence, the probiotic culture should be able to aggregate and colonize in the gut for sustaining health promoting effect. Lp9 isolate showed a relative autoaggregation of approximately 65% and 75% of the auto-aggregation potential of the standard probiotic cultures *L. johnsonii* LA1 and *L. acidophilus* LA7, respectively (Table 2). The cellular aggregation helps not only in the transient colonization but also in providing a protective shield to the host system due to formation of a bacterial biofilm [54] over the host tissue. It has been suggested that cellular aggregation is important to promote the colonization of beneficial microorganisms in several ecological niches like the GI or urogenital tracts [38], [55]–[59]. Furthermore, studies have demonstrated the induction of *L. plantarum* genes during GIT passage [60], [61]. Similarly, host's immunomodulatory genes as well as mucus secretion are also induced in response to host-microbe interaction [22], [62]. Therefore, it is important that the putative probiotic isolate should be able to persist for an extended period in the host gut to deliver the beneficial effects and Lp9 seems to possess these properties required to adapt and colonize in the GI tract.

### Functional and Health Promoting Properties of Lp9 Isolate

#### Hypocholesterolemic activity

The deconjugation of major bile salts suggested that Lp9 could play a significant role in lowering the cholesterol level under *in vivo* conditions [63], [64]. The most abundant bile salts in humans are cholate, chenodeoxycholate and deoxycholate, which are normally conjugated with either glycine (75%) or taurine (25%). Bile salts are water-soluble end products of cholesterol and are synthesized in the liver. The deconjugation of bile salts leads to decreased solubility and hence lower reabsorption in the enterohepatic system, thereby, resulting in an increased demand for cholesterol as a precursor of bile salts. The amplification of full length *bsh* ORF of 975 bp (Figure 1) using *L. plantarum* specific primers, as well as the deconjugation of the major bile salts up to the studied concentration of 0.5% suggested that Lp9 possessed a functional Bsh enzyme that could help in the depletion of cholesterol in the host. The genome analysis indicated that *L. plantarum* possessed the largest number of *bsh* genes [65] and hence well placed for ameliorating the hypercholesterolemia and protection against cardiovascular diseases.

#### Antibacterial activity

Lp9 isolate of *L. plantarum* inhibited the growth of pathogens like *E. coli*, *L. monocytogenes, S. typhi S. aureus* and *B. cereus*. Previously, *L. plantarum* has been reported to strongly inhibit the growth of *L. monocytogenes*, *E. coli* and broad range of other pathogens [66]. Plantaricin N, a bacteriocin produced by *L. plantarum* KKY12, was shown to inhibit pseudomonas, *Aeromonas sobria* and *Aeromonas cavice* [67]. Our results however suggested that antibacterial activity of Lp9 could essentially be due to lowering of pH due to the production of various organic acids by Lp9 during fermentation. LAB including *L. plantarum* produce lactic acid and acetic acid, which can serve as potent antibacterial agents against pathogens like coliforms, *Salmonella* and Clostridia spp [68]–[70]. *L. plantarum* SK1 has been reported to exhibit strong antagonistic effect against pathogens due to its capability to produce organic acids [66]. Lowering of pH is a general attribute of lactic acid bacteria, and is considered beneficial in maintaining general health of GIT and female genital tract of the host. The antagonistic effect of these bacteria as well as their own survival at a given pH, however, varies considerably depending upon the species and strains.

Apart from the antagonistic effect mediated through organic acids and bacteriocins, LAB have been reported to scavenge pathogens by competitive exclusion because of their preferential binding with GIT lining mediated by some adhesion factors like mannose-specific adhesins [13], [16]. Because of the presence of putative adhesion factors like Fbp and Mub proteins, Lp9 may potentially affect the binding of pathogenic organisms to the GI tract lining by competitive exclusion, and thus help in expediting their clearance from the host. The Caco-2 cell binding assay suggested that Lp9 could efficiently bind with human enterocytes and hence possessed the potential to compete with the pathogens in the GI tract.

#### Antioxidative activity

The Lp9 isolate of *L. plantarum* was observed to score over *L. johnsonii* and *L. acidophilus* with respect to antioxidative activity in the extracellular matrix. The intracellular antioxidative activity of Lp9 was observed to be higher than in the extracellular medium. The extracellular antioxidative activity provides protection against free radicals when viable cells colonize and propagate in the host gut. On the other hand, the intracellular extract over death of microbial cells can provide protection against the free radical species. Intracellular cell free extract from *Lactobacillus* spp. SBT2028 has been shown to be a suitable substitute for vitamin E deficiency and thereby reducing the oxidative stress in rats [71]. Lin and Yen [72] reported antioxidative activity of yoghurt microorganisms such as *Streptococcus thermophilus* and *L. delbrueckii* spp. *bulgaricus* by inhibition of linoleic acid peroxidation. Kullisar & coworkers [73] showed antioxidative potential of lactobacilli, which expressed manganese superoxide dismutase capable of eliminating hydroxyl radicals. Microorganisms producing antioxidative factors have been considered to play an important role in ameliorating the aging process [74], cardiovascular diseases [75], diabetes [76] as well as ulcers of GI tract [77] and infection of urogenital tract [24]. The higher intracellular antioxidative activity of Lp9 isolate in comparison to standard probiotics suggests that it can serve well as a probiotic adjunct in scavenging free radicals.

Preliminary *in vivo* studies in rat fed on Lp9 as food supplement showed a significant decrease in the serum cholesterol level. Lp9 also showed marked changes in the expression of pro and anti-inflammatory factors in Caco-2 cell line, which suggested the role of Lp9 in immunomodulation (unpublished results). The results are yet to be proved conclusively by exhaustive *in vivo* and clinical studies. Nevertheless, these preliminary results showed the potential of Lp9 for exploitation in future functional dairy foods to achieve health promoting effects in the host.

### Origin of *L. plantarum* Lp9 Isolate

In this study, Lp9 was isolated from buffalo milk. *L. plantarum* is a broad-lifestyle organism [1] and is one of the few lactobacilli species that has been used in traditional and industrial food fermentation and having the capability to reside in the human and animal GI tract [78]. *L. plantarum* is basically a common gut bacterium present in mouth, intestine, jejunum or rectum of majority of the healthy individuals; however colonization can be person-dependent [79], [80]. Lp9 isolate could bind with the human colonic carcinoma derived Caco-2 cell line. The PCR assay revealed Lp9 genome containing putative adhesion genes *fbp* and *mub*, which are present in microbes of GIT origin. The MUB-domain containing Mub protein has so far been shown to be exclusively encoded by lactobacilli of GIT origin [1], [42], [52], [81], [82]. Interestingly, milk-fermenting lactobacilli like *L. bulgaricus* [4] and *L. casei* [3] contain no MUB-domain containing protein in their genome, while the genome of dairy organism *L. lactis* contains only a single MUB-domain containing protein [83].

PCR amplification of full length *bsh* gene as well as the potential of Lp9 to grow in the presence of bile and degrade them suggested that Lp9 contained functional bile salt hydrolase, an enzyme present almost exclusively in GIT-colonizing bacteria [82].

These findings suggest that Lp9 could inhabit and survive the passage through GI tract and serve as a potential indigenous probiotic culture. In countries where traditional dairy system, involving human subjects in milking and processing of milk, is in practice; there exists a high probability of cross-contamination of microorganisms between human and animal products. The source of *L. plantarum* in milk might be human fecal contamination; however it remains to be unambiguously established. Although *L. plantarum* enjoys a long history of safe use in ethnic as well as commercial probiotic preparations [9], yet safety aspects and *in vivo* studies in human for ascertaining the colonization and health promoting effect of Lp9 isolate needs further investigations.

In summary, Lp9 isolate of *L. plantarum* was screened and identified by molecular-typing methods. Physicochemical and functional properties of the isolate were studied at length. Based on various survival and colonization tests prescribed by FAO/WHO [29], Lp9 isolate of *L. plantarum* was identified as a potential lead probiotic culture. Lp9 could survive the harsh acidic pH prevalent in an adult human stomach. It was capable to deconjugate the bile salts. Lp9 also showed good *in vitro* hydrophobicity, cellular autoaggregation, and the presence of genes for mucus-binding and fibronectin-binding adherence proteins as well as bile salt hydrolase enzyme, suggesting the survival and colonization potential of Lp9 in the GI tract. Lp9 isolate also exhibited health-promoting properties like free-radical scavenging antioxidative activity and antibacterial activity against several pathogens. Our results showed that Lp9 isolate of *L. plantarum* can serve as a potential indigenous probiotic adjunct culture in the development of nutraceutical products or probiotic pills for therapeutic or prophylactic applications after proper human clinical studies.

## Materials and Methods

### Sample Collection

Human milk samples were collected from lactating mothers. The human faeces sample were collected from master and graduate degree students residing at the institute hostels of National Dairy Research Institute (NDRI), Karnal, India. All the milk and faeces samples were collected from the volunteers with an explicit and informed oral consent. Informed oral consents sufficed the requirements of the review committee of the NDRI, because i) ours was purely a microbiological study which did not involve any human genetic analysis and ii) the individuals were anonymised and their data has been analyzed and presented in such a way that individual volunteers could not be identified based on features of their microbial isolates and their biological information remained fully protected throughout the study. The study reports the Lp9 isolate of *L. plantarum* isolated from buffalo milk. The buffalo milk was collected from the experimental animal herd maintained at National Dairy Research Institute, Karnal. All the study protocols were approved by the Institutional Biosafety Committee of the NDRI. The Caco-2 cell line was purchased from the National Animal Cell Repository at National Centre for Cell Science, Pune, India.

### Screening and Propagation of Bacterial Cultures

Lactobacilli were isolated from human fecal and milk samples as well as buffalo milk. The samples collected were enriched in MRS broth. For human fecal samples, sterile swabs were put in MRS broth for enrichment at 37°C for 2–3 h followed by streaking on BCP-Lac-MRS Agar. After overnight growth, yellowish colonies typical of lactobacilli were selected for morphological examination under microscope. Pour plating was also done with dilutions of 1∶10^7^ and 1∶10^8^ and the submerged colonies were selected for morphological examination using Gram staining. The putative lactobacilli isolates were further subjected to catalase test and molecular typing methods. The standard bacterial cultures namely *Lactobacillus johnsonii* LA1 and *Lactobacillus acidophilus* LA7 were kindly provided by Prof. K. J. Heller, Federal Research Institute for Nutrition and Food, Kiel, Germany; while *Lactobacillus plantarum* CSCC5276 was kindly provided by Prof. N. P. Shah, Victoria University, Australia. The pathogens, *Escherichia coli, Listeria monocytogenes, Staphylococcus aureus, Salmonella typhi*, and *Bacillus cereus*, maintained at National Collection of Dairy Cultures, National Dairy Research Institute, Karnal (India) were used in this study. Before using the pathogens as indicator organisms for testing antibacterial activity of Lp9, the cultures were propagated in BHI broth and incubated at 37°C for overnight.

### Identification of Lactobacilli Isolates by PCR

The genomic DNA from the cultures grown at 37°C for 16–18 h in MRS broth was extracted by following the protocol as described by Pospiech and Neumann [84]. The PCR reaction was carried out in a total volume of 50 µl containing forward and reverse primers, Taq DNA polymerase, DNA template and PCR buffer containing Tris-HCl, KCl, MgCl_2_ and each of the dNTP. The PCR reaction was carried out in a thermal cycler (Eppendorf Mastercycler Gradient 5331, Germany) by programming the cycling profile consisting of an initial denaturation step of 5 min at 95°C followed by an amplification for 35 cycles with denaturation (30 sec at 95°C), annealing for 30 sec at the temperatures given in Table 1 for the corresponding set of primers, and final extension of 10 min at 72°C. The PCR amplified DNA fragments were resolved by agarose electrophoresis and stained with ethidium bromide (0.5 µg/ml) followed by visualization under UV light.

Genus specific PCR assays were carried out using forward primer LbLMA-1 and reverse primer R-161 [85]. For carrying out 16S rRNA sequencing, the genomic DNA was isolated and a ∼1.4 kbp DNA fragment was amplified using high-fidelity PCR polymerase followed by bidirectional sequencing (Chromus Biotech Pvt. Ltd., India). The sequence data was aligned and analyzed using BLAST server available at http://www.ncbi.nlm.nih.gov/. Further confirmation of Lp9 isolate was carried out by PCR using the *Lactobacillus plantarum* specific primer pair Lpla3/Lpla2 as well as primers LpBSHF/R and LpBSHF1/R1 targeted against *bsh* gene encoding bile salt hydrolase. The sequence of primers has been given in Table 1.

### PCR Assay for Surface Protein Genes

Presence of putative mucus-binding protein and fibronectin-binding protein genes, *mub* and *fbp* respectively, were detected by employing the species-specific primers in a PCR assay. Two sets of forward and reverse primers were designed against the Mub gene of *L. plantarum* WCFS1 [1] using genomic segment 5/11 (accession No: AL935256.1). The primer pair LpMubD1F/R was targeted to MUB-domain (bases 2600–6200 in the *mub* ORF length of 1–6660 bp) of Mub protein lp_1643 [42], while primer pair LpMubNF/R was targeted to N-terminal part of the protein (927–2567 bp of ORF). The primer pair LpFBPF/R against the fibronectin-binding protein *fbp* gene was designed using the *L. plantarum* WCFS1 genomic sequence segment 6/11 (accession no: AL935257.1). The sequences of various set of primers used in the study are given in Table 1.

### Probiotic Attributes

To ascertain the probiotic attributes of Lp9, the isolate as well as standard cultures *L. johnsonii* LA1 and *L. acidophilus* LA7 were subjected to an array of tests as per FAO/WHO, [29] guidelines and the same have been described below.

### Acid Tolerance

MRS broth was used to simulate acidic conditions of gut after adjusting to different pH values namely 1.5 and 2.0 with 1.0 N HCl. Another set of broth was adjusted to neutral pH (7.0) to serve as a control. The broth tubes adjusted at different pH values were inoculated (at 10^9^ cfu/ml) with overnight grown cultures of lactobacilli and incubated at 37°C for 24–48 h. One ml of culture was taken from each tube immediately (0 h) and 10-fold serial dilutions were prepared in 0.1% peptone water. Pour plating was done using BCP-Lac MRS agar. Similarly, one ml of culture was taken from each tube after an interval of 1, 2 and 3 h followed by plating. The plates were incubated at 37°C for 24 to 48 h and the colony forming units (cfu) were counted.

### Bile Salt Tolerance and Bile Salt Hydrolase Activity

#### Bile salt tolerance

Fresh culture of Lp9 was inoculated in MRS broth supplemented with 1.5% and 2.0% (w/v) bile salts (Oxgall, HiMedia, India) followed by incubation at 37°C. Aliquots were withdrawn at 0, 1 and 2 h interval and plated on MRS agar at 37°C for 24 hours. Bile tolerance was assessed in terms of viable colony counts after the aforesaid incubation at 37°C.

#### Bsh activity

A direct plate assay method was employed for detection of bile salt hydrolase (Bsh) activity. Bsh activity was examined by streaking overnight grown culture of Lp9 on MRS agar containing biles like sodium deoxycholate (DCA), sodium glycocholate (GCA) and sodium taurodeoxycholate (TDCA). The petri plates were then anerobically incubated at 37°C for three days. Bsh activity was indicated when the hydrolyzed products of the salts, viz. cholic acid or deoxycholic acid, precipitated in the agar medium in and around the spots.

### Cell Surface Hydrophobicity

The bacterial adhesion to hydrocarbons was determined by following the method of Rosenberg et al. [49] with slight modification to measure the cell surface hydrophobicity. The bacterial cells grown in MRS broth at 37°C for 18 h were centrifuged and the cell pellet was washed twice with phosphate urea magnesium (PUM) buffer. The washed pellet was resuspended in PUM buffer and the absorbance was adjusted to ∼0.7 OD at 600 nm. Lactobacilli cell suspension (3.0 ml) and n-hexadecane or xylene (1.0 ml) were mixed by vortexing and incubated at 37°C for 10 min for temperature equilibration. The mixture was again briefly vortexed and incubated at 37°C for 1 h for phase separations. The aqueous phase was gently taken out to measure its absorbance at 600 nm. The surface hydrophobicity (%) was calculated as percent decrease (ΔAbs×100) in the absorbance of the aqueous phase after mixing and phase separations relative to that of original suspension (Abs_Initial_) as follows:
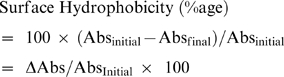
(2)


### Cell Aggregation

The freshly grown bacterial cells in MRS broth at 37°C were harvested (step 1) and the cell pellet washed twice with PBS and resuspended again in PBS to an absorbance of ∼0.5 at 600 nm (Abs_initial_). The suspension was centrifuged and the pellet was resuspended in equal volume of broth removed at step 1. The mixture was allowed to stand at 37°C for 2 h. Thereafter, 1.0 ml of the upper suspension was taken to measure the absorbance (Abs_final_) by using broth as reference. The percent difference between the initial and final absorbance would give an index of cellular autoaggregation that can be expressed as follows [10], [38]: 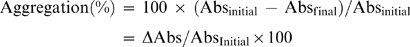
(3)


### Caco-2 Cells Adhesion Assay

Adhesion of isolates was assayed as per the method described by Jacobsen and coworkers [32]. Initially 10^5^ Caco-2 cells were seeded in each well of six-well tissue culture plates. The Dulbecco's modified Eagle's minimal essential medium (DMEM) supplemented with 10% (v/v) heat-inactivated (30 min, 56°C) fetal bovine serum, 100 U/ml penicillin, and 100 µg/ml streptomycin was used for culturing. The medium was changed with fresh medium every alternate day. Adhesion assay was done after 20 days of post confluency. The cells were then washed twice with 3 ml phosphate-buffered saline (pH 7.4). Two ml of DMEM without serum and antibiotics was added to each well and incubated at 37°C for 30 min. Approximately 10^9^ cfu/ml bacterial culture was suspended in 1.0 ml DMEM medium (without serum and antibiotics) and added to different wells. The plate was incubated at 37°C for 2 h in the presence of 5% CO_2_/95% air atmosphere. The monolayer was washed with sterile PBS and the cells were detached by trypsinization. One ml of 0.25% Trypsin-EDTA solution (Sigma Chem. Co) was added to each well of six-well plate which was then incubated for 15 minutes at room temperature. The cell suspension was platted on MRS agar by serial dilution for determining the adherent bacterial cells. The plate was incubated for 24–48 h at 37°C and colonies were counted. Bacterial cells initially added to each well of six-well plates were also counted by serial dilution and plating on MRS agar. The results of the adhesion assay were expressed as adhesion percentage, the ratio between adherent bacteria and added bacteria per well. Three independent experiments (*n* = 3) with two replicates in each experiment with Caco-2 cells of same passage were carried out.

### Antibacterial Activity

The antagonistic effects of culture supernatants of Lp9 isolate on indicator organisms like *E. coli*, *L. monocytogenes, S. typhi*, *S. aureus* and *B. cereus* were tested by the agar-well-diffusion assay. The culture supernatant of Lp9 was prepared by growing the cultures in MRS broth for 16–18 h at 37°C and removing the cells by centrifugation at 8,000xg for 5 minutes. For testing the antibacterial activity, one part of the supernatant was used at the final pH of the spent broth, while another part was neutralized with 1.0 N NaOH to pH 6.5 before setting the activity. All the solutions were sterilized by filtering through a 0.22 mm pore size cellulose acetate filter membrane before adding in to the wells. Prepoured MRS agar plates were overlaid with 10 ml of soft MRS agar inoculated with 0.3 ml of an overnight culture of the indicator organism grown in M17 broth at 37°C. After allowing the media to solidify at room temperature for 15 min, wells of 7 mm diameter were made with a sterile cork borer. The wells were sealed with one drop of soft agar and 100 µl of the cell-free supernatant was filled into each well. The plates were kept undisturbed for few hours so that supernatant can diffuse in the agar. Afterwards plates were kept at 37°C for 10–12 h and the diameter of the zone of inhibition was measured.

### Antioxidative Activity

Antioxidative activity was measured by the ABTS [2,2′-Azinobis(3-ethylene benzothiazoline) 6-Sulphonicacid)] method [86], [87]. The ABTS solution was prepared by mixing 88 µl of 140 mM potassium persulphate with 5 ml of 7 mM ABTS solution and incubating overnight in dark bottles. An aliquot of 200 µl of this solution was added to 15 ml PBS to adjust the absorbance at 734 nm to 0.7±0.02. An aliquot of 10 µl of cell supernatant was added to 1.0 ml ABTS in PBS solution in an optical cuvet and kinetics of the reaction was measured by a change in the absorbance at 734 nm. The progress curves were analyzed by the least-squares method to determine the rate constant using equation 4:
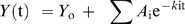
(4)where, *Y* is the signal at any time (*t*), *Y*_o_ is the signal value when no further change is observed, *k_i_* is the apparent rate constant, and *A_i_* is the total amplitude of the *i* ^th^ kinetic phase.
